# Intradermal fractional-dose inactivated polio vaccine (fIPV) adjuvanted with double mutant Enterotoxigenic *Escherichia coli* heat labile toxin (dmLT) is well-tolerated and augments a systemic immune response to all three poliovirus serotypes in a randomized placebo-controlled trial

**DOI:** 10.1016/j.vaccine.2022.03.056

**Published:** 2022-04-26

**Authors:** Jessica W. Crothers, Elizabeth Ross Colgate, Kelly J. Cowan, Dorothy M. Dickson, MaryClaire Walsh, Marya Carmolli, Peter F. Wright, Elizabeth B. Norton, Beth D. Kirkpatrick

**Affiliations:** a Department of Pathology and Laboratory Medicine, Vaccine Testing Center, University of Vermont Larner College of Medicine, Burlington, VT, USA; b Microbiology and Molecular Genetics, Vaccine Testing Center, University of Vermont Larner College of Medicine, Burlington, VT, USA; c Department of Pediatrics, Vaccine Testing Center, University of Vermont Larner College of Medicine, Burlington, VT, USA; d Department of Pediatrics, Geisel School of Medicine at Dartmouth, Hanover, NH, USA; e Department of Immunology and Microbiology, Tulane University, New Orleans, LA, USA

**Keywords:** Polio, Vaccine, Injectable, Adjuvant, Mucosal Immunity

## Abstract

Eradication of poliomyelitis globally is constrained by fecal shedding of live polioviruses, both wild-type and vaccine-derived strains, into the environment. Although inactivated polio vaccines (IPV) effectively protect the recipient from clinical poliomyelitis, fecal shedding of live virus still occurs following infection with either wildtype or vaccine-derived strains of poliovirus. In the drive to eliminate the last cases of polio globally, improvements in both oral polio vaccines (OPV) (to prevent reversion to virulence) and injectable polio vaccines (to improve mucosal immunity and prevent viral shedding) are underway. The *E. coli* labile toxin with two or “double” attenuating mutations (dmLT) may boost immunologic responses to IPV, including at mucosal sites. We performed a double-blinded phase I controlled clinical trial to evaluate safety, tolerability, as well as systemic and mucosal immunogenicity of IPV adjuvanted with dmLT, given as a fractional (1/5th) dose intradermally (fIPV-dmLT). Twenty-nine volunteers with no past exposure to OPV were randomized to a single dose of fIPV-dmLT or fIPV alone. fIPV-dmLT was well tolerated, although three subjects had mild but persistent induration and hyperpigmentation at the injection site. A ≥ 4-fold rise in serotype-specific neutralizing antibody (SNA) titers to all three serotypes was seen in 84% of subjects receiving fIPV-dmLT vs. 50% of volunteers receiving IPV alone. SNA titers were higher in the dmLT-adjuvanted group, but only differences in serotype 1 were significant. Mucosal immune responses, as measured by polio serotype specific fecal IgA were minimal in both groups and differences were not seen. fIPV-dmLT may offer a benefit over IPV alone. Beyond NAB responses protecting the individual, studies demonstrating the ability of fIPV-dmLT to prevent viral shedding are necessary. Studies employing controlled human infection models, using monovalent OPV post-vaccine are ongoing. Studies specifically in children may also be necessary and additional biomarkers of mucosal immune responses in this population are needed.

Clinicaltrials.gov Identifer: NCT03922061.

## 1 Introduction

In 1988, approximately 125 countries were endemic for polio and 350,000 children annually suffered from paralytic disease [1], [2]. Global eradication of polio has been a major initiative of the World Health Organization (WHO) with successful prevention of 16 million cases of paralytic disease in the past 30 years, the declaration of Africa as wild-type polio free in August 2020, and subsequent reduction in wild-type poliovirus endemicity to only 2 countries: Pakistan and Afghanistan [3]. Advances to date have been largely attributable to the use of multiple doses of the trivalent Sabin live oral polio vaccine (tOPV), which stimulates robust systemic and mucosal immunity to poliovirus in children [4].

At this stage in the Polio Endgame, however, final eradication is challenged by the ongoing use of OPV. Once shed into the environment following oral vaccination, OPV live viruses have the ability to revert to neurovirulence as circulating vaccine-derived polioviruses (cVDPV), which can infect those with incomplete polio-specific immunity and cause vaccine-associated paralytic polio (VAPP) [2], [5], [6]. Environmental surveillance and case monitoring for patients with flaccid paralysis demonstrates ongoing cVDPV emergence in numerous countries in Africa and Asia where OPV is still used [7].

To achieve final eradication, the methodical withdrawal of OPV began under WHO guidance in April 2016 with an ultimate goal of global transition to IPV-only vaccination. Trivalent OPV (tOPV) was replaced by bivalent OPV (bOPV, OPV 1 + 3) plus at least one dose of injectable trivalent inactivated polio vaccine (tIPV) [8], [9]. Combined vaccination schedules with bOPV and tIPV have demonstrated similar mucosal immune responses to poliovirus serotypes 1 and 3 when compared to OPV-only regimens; however without inclusion of OPV2 mucosal responses to serotype 2 are diminished [10], [11], [12], [13]. Previous studies suggest that IPV alone may be insufficient to induce the protective mucosal immunity necessary to limit vaccine virus shedding and thus reduce environmental transmission in populations where cVDPD persists, particularly without initial priming with a live virus vaccine, such as OPV [14], [15], [16]. Consequently, during the transition to IPV-only schedules, children may have a gap in mucosal immunity, particularly to serotype 2, allowing shedding following poliovirus exposure (wild-type or vaccine derived) and transmission to unvaccinated or otherwise unprotected or immunocompromised children [10], [17].

To facilitate the complete removal of OPV and final polio eradication, an effective adjuvanted IPV which stimulates mucosal immune responses and curtails poliovirus shedding and transmission would be ideal [20]. The double mutant [LT(R192G/L211A)] Enterotoxigenic *Escherichia coli* heat labile toxin (dmLT) adjuvant has been extensively studied for safety and systemic and mucosal immunogenicity in animals and humans both alone and admixed with other (non-IPV) vaccines by oral, sublingual, intramuscular and intradermal routes [18], [19], [20], [21], [22], [23], [24], [25]. Derived from an enteric bacteria (*Escherichia coli*), dmLT appears to have a unique ability to stimulate mucosal immune responses in pre-clinical models, even when admistered at anatomically distant sites [20], [26], [27], [28].

At the same time, significant shortages of IPV have been problematic and have constrained eradication efforts and the safety net against cVDPV infection [29]. Similar issues may arise during the period of containment after complete withdrawal of OPV or during isolated cVDPV outbreaks. To stretch IPV supplies, fractional doses (fIPV) at one-fifth of the full dose of trivalent vaccine, can be administered intradermally. This approach, now endorsed by the WHO for use into routine immunization activities as well as in outbreak responses and supplementary immunization activities, has been shown to be a safe and effective way to stimulate systemic immune responses comparable to a full dose of IPV [30]. Countries that have begun to adopt fIPV into routine immunization programs include India, Nepal, Cuba, Bhutan, and Ecuador.

Toward finding a solution to both the mucosal immune response issue and the need to extend IPV supplies, we evaluated the safety, reactogenicity and immunogenicity of intradermal fIPV adjuvanted with dmLT (fIPV-dmLT) in a first-in-humans phase I randomized, double-masked, placebo-controlled clinical trial including volunteers with no past exposure to OPV.

## 2 Methods

### 2.1 Study design and participants

A phase 1, randomized, double-masked, placebo-controlled clinical trial to evaluate the safety, reactogenicity and immunogenicity of intradermal fractional dose inactivated polio vaccine adjuvanted with Enterotoxigenic *Escherichia coli* heat labile toxin (fIPV-dmLT) was performed at a single site in Burlington, Vermont (USA). Eligible participants were healthy adults aged 18–45 years with no history of oral polio vaccination (by review of vaccine records) and no history of anaphylaxis, Guillian-Barre syndrome, receipt of a live vaccine within 28 days or a killed vaccine within 14 days, receipt of blood products within 6 months, and no history of hypersensitivity to any component of IPV. Pregnant and lactating women were excluded, and study participants of child-bearing potential were required to use effective contraception for the first 28 days of the study.

This trial was approved by the United State Food and Drug Administration’s (FDA) Investigational New Drug program (IND#18511) and by the Institutional Review Board (IRB) at the University of Vermont (UVM). This study was conducted in compliance with the ethical principles of the Declaration of Helsinki. All participants provided written, informed consent prior to initiation of any study-related activities.

### 2.2 Randomization and masking

Study participants were randomized 2:1 to receive one fractional (1/5th) dose of trivalent IPV with or without 0.47 µg of dmLT adjuvant administered by intradermal (ID) injection over the deltoid area following standard procedures. Treatment assignments were generated by the study statistician using block randomization and assigned sequentially at study enrollment using the pre-generated list. The study statistician was not otherwise involved in trial conduct. Participants and study personnel responsible for clinical evaluations or data generation were masked to treatment arm assignment. Vaccines were prepared and administered by unmasked study personnel with no other study involvement.

### 2.3 Investigational product

Sanofi’s licensed IPOL trivalent inactivated polio vaccine (tIPV; NDC 49281860-78) was delivered intradermally in the upper arm (deltoid region) at the dose-sparing fractional volume of 1/5 the full dose (0.1 mL) with or without 0.47 µg of dmLT adjuvant per dose. The dose of 0.47 µg was chosen following preclinical toxicology assessments of intradermal dmLT in combination with IPV which showed this dose to be both immunogenic and well-tolerated in animal models (unpublished data). Additionally, preclinical and clinical work assessing the use of dmLT as an injectable adjuvant for Enterotoxigenic *Escherichia coli* (ETEC) vaccines has similarly found doses of 0.5 µg to be immunogenic with limited local reactogenicity [22], [31]. dmLT was produced according to good manufacture practice (GMP) specification by IDT Biologika Corporation and supplied by PATH in the form of 500 μg lyophilized cake in 3 mL vials (lot 001-0816) and maintained at −20 °C during transport and storage at the clinical site for up to 12 months prior to use. dmLT was rehydrated with 0.5 mL of sterile water for injection to achieve a final concentration of 1 mg/ml dmLT. The adjuvanted IPV vaccine was prepared by diluting rehydrated dmLT adjuvant with IPOL® vaccine on the day of vaccination by the Research Pharmacy at the University of Vermont Medical Center to produce a solution containing 47.6 µg/mL dmLT in IPOL® IPV. Doses of 0.1 mL of the final admixture were drawn into individual 3 mL syringes with 26 gauge needle for single-subject administration within 15 min of admixing. Dose verification was performed to confirm potency of the product. For the control arm, 0.1 mL IPOL was prepared in individual syringes identical to the investigational product and dosed within 15 min.

### 2.4 Procedures

Study participants were dosed per randomization on Day 0 with in-person follow-up visits for safety evaluation and specimen collection at Days 1, 7, 10 and 28. Blood specimens were collected for safety labs and immunologic endpoints at every in-person visit. Stool specimens were collected on Days 0 and 28 for evaluation of mucosal immune response. Participants maintained a daily surveillance diary to track adverse events (AEs) through Day 7, including twice daily temperature monitoring. Any unresolved AEs at Day 7 were followed until resolution. Following Day 28, participants received monthly phone calls for safety follow-up to one year.

### 2.5 Safety monitoring

Safety monitoring was performed at all scheduled and unscheduled (ad hoc) study visits, via daily participant surveillance dairies, and on monthly phone calls from Day 28 through one year. Safety-related procedures included physical examinations at screening and on dosing day, pregnancy testing of female participants of childbearing potential at Day 0 (prior to vaccination), vital signs, medical history and concomitant medications, and injection site exams. Clinical laboratory assessments consisted of hematology, complete blood count, and a comprehensive metabolic panel at screening and Day 7. Solicited adverse events included systemic reactogenicity (fever, rash, fatigue, nausea, vomiting, anorexia, diarrhea, myalgia, arthralgia, rash, headache) and injection site reactogenicity (pain, erythema, tenderness, induration, pruritis, edema, and hypo/hyperpigmentation). Hypo/hyperpigmentation was defined as an observed color change and graded by the size of the affected region (grade 1, 0–20 mm; grade 2, 21–50 mm; grade 3, ≥50 mm). Unsolicited AEs were also captured. All AEs were graded for severity and assessed for relationship to the study vaccine, action taken and outcome. Serious adverse events (SAEs) were captured per the standard 21 CFR 312.32 definition. Procedures were in place to report all SAEs to the University of Vermont IRB, Independent Safety Monitor (ISM), and the U.S. Food and Drug Administration (FDA), as well as to report medically attended adverse events (MAAEs) to the FDA.

An interim safety analysis was performed by an unmasked biostatistician and separately by the Independent Safety Monitor (ISM) after an initial cohort of 10 participants reached Day 7 post-vaccination and following completion of all Day 28 follow-up visits. Halting rules for unacceptable vaccine reactogenicity were articulated in the protocol.

### 2.6 Immunogenicity assessments

Systemic immunogenicity was determined by serum neutralizing antibody (SNA) titers to poliovirus types 1–3 at baseline (Day 0) and 7, 10, and 28 days following vaccination. Antibody titer assays were performed at the US Centers for Disease Control and Prevention Polio and Picornavirus Laboratory Branch using a WHO-standardized 7-day plaque assay. Neutralization titers were determined by measuring cytopathic effect of inoculated HEP-2C cells using Vero cell suspensions and type 1,2,3 Sabin virus strains in combination with serial dilutions of subject sera specimens [32]. Standard measures of positive antibody titers (≥1:8) and antibody boosting (≥4-fold rise) were used.

Mucosal immunogenicity was measured by fecal neutralizing antibody titers (fNAB) and serotype-specific fecal IgA assays at baseline (Day 0) and Day 28 following vaccination (laboratories of Dr. Peter Wright, Dartmouth Hitchcock Medical Center, Lebanon, N.H. and Dr Margaret Ackerman, Thayer School of Engineering, Dartmouth College, Hanover, NH). These timepoints were chosen based upon previous work which showed a sustained and continued raise in fecal antibody responses to poliovirus through week 4 following OPV exposure [33]. fNAB titers were assessed using polio non-replicating pseudoviruses comprising luciferase-encoding replicons with polio capsid proteins (derived from all 3 polioviruses) by previously reported methods [33]. To evaluate fecal serotype-specific IgA, a customized multivariate Luminex assay was developed in which carboxylated beads were covalently coupled to inactivated polioviruses or anti-human IgA. Assay readout was determined by subtracting background signal from mean florescent intensity (MFI) and converting to a serum IgA-equivalent concentration based on a standard curve.

### 2.7 Endpoints and statistical analyses

The primary endpoint was vaccine-related adverse events (AEs), graded by severity, occurring within 28 days of dosing. The primary outcome measure was the frequency of systemic and injection site AEs in each trial arm. The secondary safety endpoint was the proportion of participants with at least one serious adverse event occurring within 28 days of vaccine administration. Systemic immunogenicity was a secondary outcome measured by the proportion of participants with ≥4-fold boost in polio SNA from Day 0 to Day 28. Mucosal immunogenicity outcomes (fNAB and serotype-specific fecal IgA) were exploratory.

Descriptive statistics were used to summarize demographic and baseline characteristics. As a first-in-humans phase I study, no formal sample size calculation was performed. Safety and immunogenicity data were analyzed using summary statistics. AEs are presented by severity and treatment arm, with primary outcome (safety) analysis by Intention to Treat with one-sided Fisher’s Exact tests for differences between treatment groups. The proportion of participants meeting serum neutralizing antibody boosting criteria was compared between treatment arms by 2-sided Fisher Exact test. Quantitative serum neutralizing antibody titer and peak change in titer was compared between treatment arms by t-tests. Fecal neutralization and fIgA responses were analyzed quantitatively with comparisons made between groups at each timepoint (Day 0 and Day 28) as well as change over time by Mann Whitney *U* test. The proportion of subjects with fecal neutralization was compared by Fisher Exact test. Spearman’s Rank Correlation was used to evaluate the relationship between fecal IgA and fecal neutralizing antibodies. Data analysis was performed using R 4.0.2 and SAS 9.4 software (SAS Institute Inc., Cary, North Carolina, United States).

## 3 Results

### 3.1 Participant demographics

**Fig. 1:**
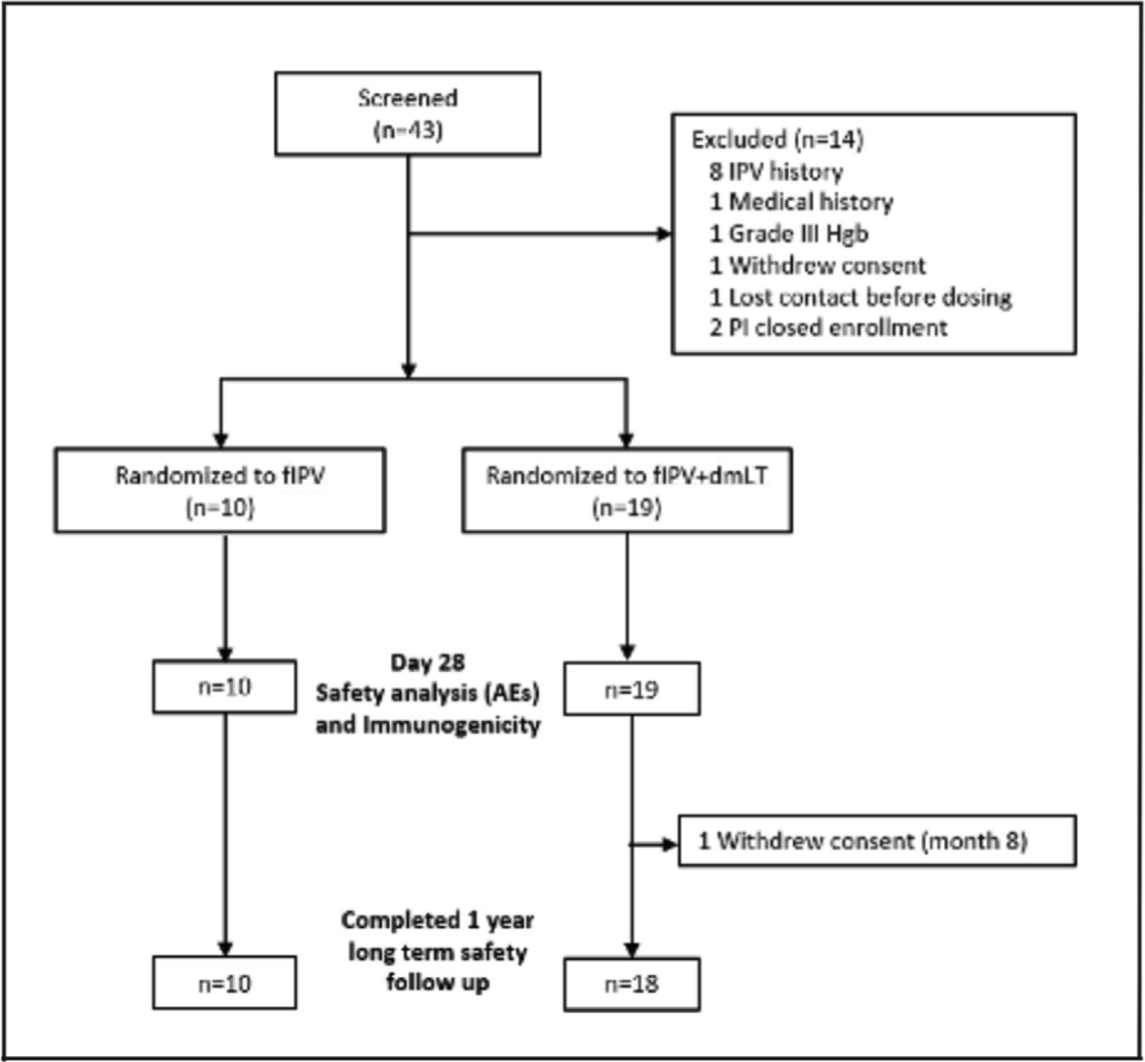
Diagram of study participation.

Twenty-nine (29) participants were enrolled between March and June 2019. Nineteen (19) participants received fIPV adjuvanted with dmLT (fIPV + dmLT) and 10 participants received fIPV alone, see Fig. 1 for a flow diagram of study participation. Vaccine was delivered by intradermal injection in both groups. The mean age at enrollment was 18.8 years (±0.6 SD) in the fIPV arm and 19.0 years (±0.7 SD) in the fIPV + dmLT arm (range for both arms was 18–20 years). In the fIPV + dmLT arm, 63% of participants were female (n = 12) and 90% (n = 9) of fIPV-only recipients were female. Table 1 summarizes race and ethnicity data for the enrolled population.

**Table 1:** Demographic Data for the Enrolled Population.

**N (column %)**	**fIPV only (n = 10)**	**fIPV + dmLT (n = 19)**	**All (N = 29)**
Sex Male Female	1 (10) 9 (90)	7 (37) 12 (63)	8 (28) 21 (72)
Ethnicity Non-Hispanic Hispanic	9 (90) 1 (10)	17 (89) 2 (11)	26 (90) 3 (10)
Race Black/African American White Other	1 (10) 9 (90) 0	0 18 (95) 1 (5)	1 (3.5) 27 (93) 1 (3.5)
Age group (years) 18 19 20	3 (30) 6 (60) 1 (10)	4 (21) 11 (58) 4 (21)	7 (24) 17 (59) 5 (17)
Age (years) Mean (SD) Median (IQR) Min, Max	18.8 (0.6) 19 (18–19) 18, 20	19 (0.7) 19 (19–19) 18, 20	18.9 (0.6) 19 (19–19) 18, 20
Weight (lbs) Mean (SD) Median (IQR) Min, Max	140.8 (14.9) 141.8 (129.5–148.8) 119.8, 168.8	149.6 (26.5) 140.2 (126.8–163.1) 118.2, 216.2	146.6 (23.3) 140.6 (129.5–154.6) 118.2, 216.2
BMI Mean (SD) Median (IQR) Min, Max	23.0 (2.7) 22.9 (22.0–24.7) 18.8, 27.7	23.5 (4.2) 21.8 (20.9–24.5) 19.0, 36.1	23.3 (3.7) 22.4 (20.9–24.5) 18.8, 36.1

### 3.2 Safety

Twenty-eight participants completed all follow-up assessments. All solicited AEs were mild, except one instance each of moderate, self-limited myalgia and arthralgia reported in the same volunteer on Day 3 post vaccination and lasted two days. There was no difference in systemic AEs related to the vaccine between treatment arms through Day 28 following vaccination (Table 2) and all systemic AEs resolved by Day 28 post-vaccination.

**Table 2:** Adverse events (AE) possibly, probably, or definitely related to vaccine, through Day 28 post-vaccination.

**Adverse Event (AE)** **Severity**	**Treatment Arm**		***P*-value**^1^
	**fIPV (N = 10)** **n (%)**	**fIPV + dmLT (N = 19)** **n (%)**	
**Systemic AEs**			
Fatigue	1 (10.0)	4 (21.1)	0.424
Headache	1 (10.0)	3 (15.8)	0.571
Anorexia	0	2 (10.5)	0.421
Myalgia	1 (10.0)	1 (5.3)	0.889
mild	1	0	
moderate	0	1	
Arthralgia	1 (10.0)	1 (5.3)	0.889
mild	1	0	
moderate	0	1	
Fever	0	1 (5.3)	0.655
Diarrhea	0	1 (5.3)	0.655
Nausea	0	0	N/A
Vomiting	0	0	N/A
Rash	0	0	N/A

**Local reactogenicity AEs**			
Erythema	10 (100.0)	17 (89.4)	1.000
Tenderness	7 (70.0)	16 (84.2)	0.330
Induration	5 (50.0)	18 (94.7)	0.010
Hyperpigmentation^2^	2 (20.0)	14 (73.7)	0.008
Pruritus	0	8 (42.1)	0.017
Pain	1 (10.0)	2 (10.5)	0.733
Rash at Injection site	0	1 (5.3)	0.655
Hypopigmentation	0	0	N/A
Edema	0	0	N/A

**Unsolicited AEs**			
Upper arm muscle pain	2 (20.0)	0	1.000
Shoulder joint pain	0	1 (5.3)	0.655
Bruising at injection site	1 (10.0)	1 (5.3)	0.889
Desquamation	0	2 (10.5)	0.421

All AEs were mild, except where noted.

1 One-sided *P*-value Fisher’s Exact test for increased incidence in fIPV + dmLT arm.

2 One additional instance of hyperpigmentation with onset at study Day 36 in the fIPV + dmLT group is not included in the table.

Among local reactogenicity AEs, induration, hyperpigmentation and pruritis were more frequent in the fIPV + dmLT arm (*p* = 0.010 induration, *p* = 0.017 pruritus and *p* = 0.008 for hyperpigmentation) (Table 2). Three subjects in the fIPV-dmLT arm reported mild (grade 1) hyperpigmentation at the final one-year visit. No SAEs or medically attended adverse events (MAAE) related to the investigational product were identified or reported in either treatment arm during the one-year follow-up period.

### 3.3 Immunogenicity

**Fig. 2:**
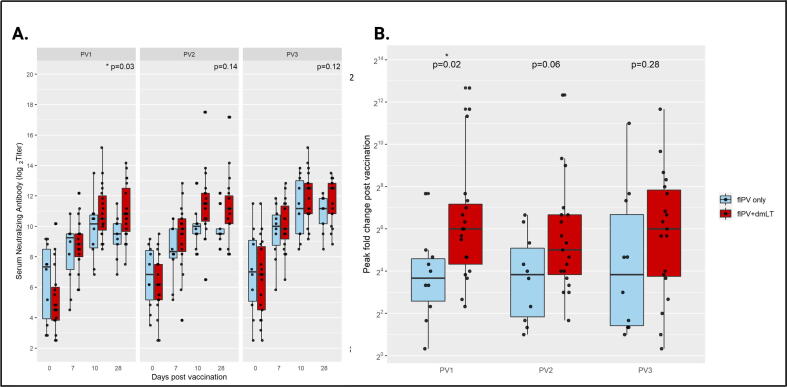
**Serum Neutralizing Antibody Titers.** Serum neutralizing antibody (SNA) titers by treatment group. Black dots represent results from individual participants. Mean serotype-specific SNA are provided by day with comparisons between groups made at Days 0 (baseline) and 28 (A). Peak rise in serotype-specific SNA (B). Comparisons between groups are made by *t*-test and p-values < 0.05 are considered to be statistically significant.

The immunogenicity endpoint for this study, ≥4-fold rise in polio-specific SNA from baseline (Day 0) to any time point post-vaccination up to Day 28, was achieved in 28 of the 29 participants for at least one serotype and for all three serotypes in 72% (n = 21). While a larger proportion of subjects in the adjuvanted arm achieved a 4-fold rise in SNA to all three poliovirus strains (84% in the fIPV + dmLT arm versus 50% in the fIPV arm), the serotype-specific differences between groups did not reach statistical significance (p = 0.11 (PV1); p = 0.11 (PV2); p = 0.14 (PV3), and p = 0.08 (all serotypes). Results are presented by treatment group and poliovirus serotype in Table 3. Notably, the peak fold rise in serotype-specific SNA titers were consistently higher in the dmLT-adjuvanted group, as shown in Fig. 2. These differences between arms were statistically significant for serotype 1 (p = 0.02 (PV1).

**Table 3:** Participants with a ≥ 4-fold Rise in Serum Neutralizing Antibody Titers, by Study Group.

	Treatment Arm				p value^1^
	fIPV (n = 10)		fIPV + dmLT (n = 19)		
	<4-fold rise	≥4-fold rise	<4-fold rise	≥4-fold rise	
**PV1 (n,%)**	2 (20)	8 (80)	0	19 (100)	0.111
**PV2 (n,%)**	3 (30)	7 (70)	1 (5.3)	18 (94.7)	0.105
**PV3 (n,%)**	4 (40)	6 (60)	2 (10.5)	17 (89.5)	0.143
**All (n,%)**	5 (50)	5 (50)	3 (15.8)	16 (84.2)	0.083

1 *2-sided Fisher Exact test.*

Serum neutralizing antibody (SNA) responses were measured to all 3 poliovirus serotypes (Sabin strains 1–3) at baseline and Days 7, 10 and 28 following vaccination. At baseline, 21 of the 29 subjects were seropositive for all three polio serotypes and 28 had a titer of >1:8 for at least one serotype. Baseline seropositivity to Sabin 1 was lowest among serotypes with 79% of volunteers (23 of 29) positive versus 93% (27/29) for both Sabin 2 and 3. There was no difference in mean serotype-specific SNA titers between dosing groups at baseline. SNA titers increased in both treatment arms following vaccination with peak titers achieved at Day 10, with the exception of PV1 in the dmLT-adjuvanted group, which continued to rise to Day 28. (Fig. 2). Additionally, at Day 28, mean antibody titers were higher to all 3 serotypes in the dmLT-adjuvanted group, although this difference was only significant for PV1 (p = 0.03 (PV1), p = 0.14 (PV2), p = 0.12 (PV3)), see Fig. 2**.**

Mucosal immune response was measured by serotype specific fecal IgA (fIgA) and fecal neutralizing antibody (fNAB) titers at baseline and 28 days following vaccination. Fecal IgA was detected at baseline in all participants and total baseline fecal IgA levels were comparable between groups with 31,839 ng/mL (IQR 23,278 to 221,335) in the fIPV only arm versus 41,806 ng/mL (IQR 7,206 to 72,781) among dmLT recipients (p = 0.84, Mann Whitney *U* test). While all participants had documented serotype specific serum neutralization at baseline, stool neutralization (fNAB titer >4) was undetectable to all three serotypes in 93% (n = 27) of subjects at baseline and serotype specific fIgA to poliovirus types 1, 2, and 3 were comparably low across both groups (Supplementary Table 1).

**Fig. 3:**
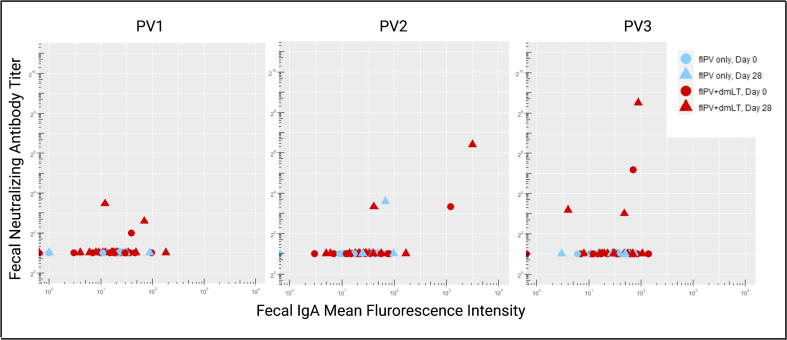
**Poliovirus Specific Stool Antibody Levels.** Pairwise correlations between poliovirus serotype specific stool IgA and serotype-specific neutralization by treatment group (fIPV, blue; fIPV + dmLT red) and day (pre-vaccination, circles; post-vaccination, triangles). Spearman’s rho: PV1 = 0.191 (p-value 0.151); PV2 = 0.383 (p-value 0.003); PV3 = 0.147 (p-value = 0.272).

At Day 28 post-vaccination, 3/19 subjects who received fIPV + dmLT had fNAB titers >4 to at least one poliovirus serotype (one each of all three serotypes), versus 1/10 subjects who received fIPV alone, serotype 2 (p = 1.00; Fisher Exact test). A correlation between serotype specific fNAB titers and fIgA levels was seen (Spearman’s rho: PV1 = 0.191 (p-value 0.151); PV2 = 0.383 (p-value 0.003); PV3 = 0.147 (p-value = 0.272) (Fig. 3). There was no difference between treatment arms in serotype specific mean fNAB titers or fIgA levels at Day 28. Similarly, there was no difference between groups in change in fIgA levels from Day 0 to Day 28 for any serotype (Supplemental Table 1). Interestingly, a single subject in the fIPV-dmLT arm showed significant boosting of serotype-specific fNAB titers following vaccination with a correlating rise in fIgA levels (data not shown).

## 4 Discussion

Despite the tremendous success of polio eradication efforts, final eradication requires novel strategies targeted at eliminating circulation and transmission of any live poliovirus, wild type or vaccine-derived. To this end, humans must stop excreting polioviruses and environmental reservoirs must be depleted. Novel polio vaccines must supply enough immune protection at muscosal surfaces to limit viral replication and shedding in the feces. Although inactivated polio vaccine (IPV) protects the recipient from symptomatic disease through systemic immunity, it does not stimulate the robust mucosal immunity necessary at intestinal sites to arrest shedding. Thus, even post-vaccination, an IPV-protected child can still shed live polioviruses, thus contining the cycle of transmission and maintaining an environmental reservoir of disease.

Evaluation of new vaccines include novel oral polio vaccines with a limited ability to revert to neurovirulence [34], [35], [36], and inactivated/injectable vaccines with adjuvants designed to stimulate mucosal immunity. Using healthy adult volunteers who had never received oral polio vaccines (OPV), we performed a double blinded, placebo-controlled clinical phase I trial of a fractional dose double-mutant labile toxin (dmLT) adjuvanted IPV vs. IPV alone. This vaccine was given intradermally at a fractional (1/5th) dose. The dermis and epidermis are extremely rich in professional antigen-presenting cells (APC) and intradermal vaccine delivery has been shown to enable dose-sparing strategies for other vaccines as well as for polio [38], [39], [40], offering an approach that could significantly extend global IPV supplies. Data suggesting that intradermal administration of antigen-adjuvant combinations can confer enhanced mucosal protection makes this approach additionally appealing. [41], [42], [43] Our findings demonstrate that intradermal fIPV adjuvanted with dmLT has a favorable safety profile and is capable of eliciting a robust systemic immune response in healthy, OPV-naïve young adults.

Our primary endpoint was safety and no significant or serious adverse events (AE) with the addition of intradermal dmLT to fIPV were identified. This adds to prior clinical data which has shown a favorable safety profile for oral, sublingual, intramuscular, and intradermal administration of dmLT both alone and in combination with various antigens [22], [23], [24], [44]. While a degree of increased local reactogenicity was observed, including mild induration, hyperpigmentation, and pruritis at the injection site, these were not unexpected. Prior pre-clinical work has revealed erythema and dermal induration with increasing doses of intradermally-administered dmLT in various animal models [28]. Here, while pruritis resolved relatively quickly in the dmLT group, both mild induration and mild hyperpigmentation persisted for a mean of 36 and 131 days, respectively.

We demonstrate that dmLT is capable of stimulating an enhanced systemic immune response when combined with fIPV: the dmLT-IPV vaccine prompted increased levels of serum neutralizing antibody (SNA) responses to all three serotypes compared to fIPV alone. While we failed to find a statistically significant difference in the proportion of participants who achieved a 4-fold rise in SNA, dmLT induced higher levels of SNA to all 3 serotypes at Day 28, including a statistically significant increase to PV1. This finding is particularly significant, as PV1 remains the only wildtype poliovirus serotype still in circulation [45]. These results support further investigation of the use of dmLT as a vaccine adjuvant, particularly in combination with fractional dose IPV in order to extend global supplies of IPV.

Regarding the impact of dmLT-IPV on markers of mucosal immunity, we found extremely low-to-undetectable levels of serotype-specific fecal IgA and fecal neutralizing antibody in the vast majority of participants, both before and after vaccination in both IPV and dmLT-IPV vaccinated groups. Poliovirus-specific fecal antibodies are known to occur following OPV exposure in infants and appear to correlate with fecal shedding dynamics [10], [33], but the ability of IPV-based vaccination regimens to induce intestinal antibody responses is less clear. While a report by Brickley *et al* revealed detectable levels of virus-specific fecal antibodies in IPV-vaccinated infants [46], a more recent study showed limited enteric antibody responses in IPV-exposed adults, even following OPV challenge [47], suggesting that fecal antibodies may not be a suitable correlate of intestinal immune responses in adult populations. The reasons for this are unclear but may reflect age-related differences in the mucosal immune response [48], [49], [50], [51]. Nevertheless, it remains unclear whether dmLT-IPV would have had a more demonstrable impact on markers of mucosal immunity in children, the population of highest interest. For example, the lack of a strong mucosal immune response in our study population, as measured by fecal antibodies, may be complicated by the fact that subjects have already been primed with tIPV in infancy. While dmLT may be unable to redirect an anamnestic immune response in adulthood, it’s ability to enhance a mucosal response during initial antigen exposure may still be possible. Since reduction of viral replication in the gut is the critically important goal in order to limit transmission and environmental reservoirs of disease, studies are needed in children and the use of OPV as the only gold-standard “challenge” test of enteric immunity to polioviruses should be employed.

Our study was limited by its small sample size. Additionally, while we made all efforts to exclude subjects with prior exposure to OPV through review of primary vaccination records, it is possible that subjects could have been previously secondarily infected by OPV-vaccinated vaccinees, or during travel to endemic regions. We feel this is unlikely however given that OPV was removed from use in our catchment area prior to the birth of participants included in this study. The inclusion of healthy adults limits the applicability of this data in target populations, namely young children and infants, particular in low and middle income countries. The use of surrogate markers of mucosal immunity (fecal neutralizing antibodies and poliovirus-specific IgA) in lieu of actual viral shedding data upon exposure to poliovirus also limits our ability to reach meaningful conclusions regarding the impact of dmLT in stimulating mucosal immune responses, particularly in pediatric populations.

The favorable safety profile demonstrated herein, as well as dmLT’s ability to augment systemic immune responses, suggests that dmLT may have a role as a safe and effective vaccine adjuvant. This is particularly relevant when considering the ability of dmLT to functionally augment global IPV supplies through fractional dosing strategies, and the potential impact of enhanced immune responses to a single vaccine dose when combating polio outbreaks.

The ability of dmLT to direct a polio-specific immune response necessary to control polio virus replication and shedding at intestinal sufaces however, is still unclear. Collectively, we are challenged by our poor understanding of mucosal immunity and how it can be effectively stimulated in humans. Because SNA to poliovirus have proven to be a strong correlate of disease protection, it is natural to assume that neutralizing antibodies may also play an important role in limiting viral replication and shedding at mucosal surfaces. While stool-based neutralization and serotype-specific IgA antibodies assays have shown strong correlations with poliovirus shedding dynamics, levels do not appear to be predictive of future shedding, thus limiting their use as a true correlate of protection, particularly in adult populations in which levels are often low to absent. It is likely that alternative immunologic pathways are critical to establishing immunologic protection at mucosal sites where initial viral entry and replication occurs. Tissue-resident memory T cells are of particular interest, as they would be poised to respond quickly to subsequent viral infection and capable of arresting viral shedding through direct cytolytic activity. It is also possible that dmLT-IPV induced mucosal T cell responses, as has been reported in mice [26], but these were not examined in this study. The ratio of adjuvant to antigen may also have a significant impact on its ability to direct desireable immune responses and should be examined in future studies. Additional investigations are ongoing and will assess the impact of dmLT on mucosal immunity to poliovirus and seek to demonstrate reductions in viral shedding upon OPV challenge as a true marker of mucosal immunity. The ability to correlate viral shedding dynamics with other systemic and/or immunologic markers in order to better establish a reproducible correlate of mucosal immunity to poliovirus is also warranted.

While significant progress has been made in the fight to eradicate polio, annual increases in circulating vaccine-derived poliovirus (cVDPV) case counts have been observed since tOPV withdrawal in 2016 and further poliovirus transmission is expected following interruptions in polio vaccination campaign efforts during the COVID-19 pandemic [7], [52]. New tools are urgently needed to support sustained eradication efforts. This first in-human evaluation of a novel mucosal adjuvant provides reassuring evidence of the safety and immunogenicity of intradermal fIPV-dmLT and supports its continued clinical development to assist in global efforts to achieve polio eradication.

## 5 Conclusion

Fractional dose-IPV when adjuvanted with dmLT, is safe and well-tolerated in OPV-naïve experienced adults and elicits higher serotype specific neutralizing antibody titers, when compared to IPV alone. Differences in mucosal responses were not seen with use of serotype-specific fecal IgA as marker for mucosal immunity. Further work to demonstrate control of polio virus shedding is necessary, as is additional work to better understand desired post-polio vaccine immune response at mucosal surfaces.

## Declaration of Competing Interest

The authors declare that they have no known competing financial interests or personal relationships that could have appeared to influence the work reported in this paper.

## References

[1] Rutter PD, Hinman AR, Hegg L, King D, Sosler S, Swezy V, et al., Transition Planning For After Polio Eradication. J Infect Dis 2017;216:S287–92. 10.1093/infdis/jix026 PMC5853549 28838183

[2] WHO. World Health Organization Weekly Epidemiological Record. Polio Vaccines: WHO Position Paper-March 2016. In: World Health Organization Weekly Epidemiological Record. Polio Vaccines: WHO Position Paper-March 2016. https://www.who.int/wer/2016/wer9112.pdf?ua=1.

[3] Organization WH Africa eradicates wild poliovirus. In: Africa eradicates wild poliovirus. https://www.afro.who.int/news/africa-eradicates-wild-poliovirus.

[4] Faden H., Modlin J.F., Thoms M.L., McBean A.M., Ferdon M.B., Ogra P.L. (1990). Comparative evaluation of immunization with live attenuated and enhanced-potency inactivated trivalent poliovirus vaccines in childhood: systemic and local immune responses. J Infect Dis.

[5] Platt L.R., Estivariz C.F., Sutter R.W. (2014). Vaccine-associated paralytic poliomyelitis: a review of the epidemiology and estimation of the global burden. J Infect Dis.

[6] Burns C.C., Diop O.M., Sutter R.W., Kew O.M. (2014). Vaccine-derived polioviruses. J Infect Dis.

[7] Alleman M.M., Jorba J., Greene S.A., Diop O.M., Iber J., Tallis G. (2020). Update on Vaccine-Derived Poliovirus Outbreaks — Worldwide, July 2019–February 2020. Morbidity Mortal Wkly Rep.

[8] Rubin J, Ottosen A, Ghazieh A, Fournier-Caruana J, Ntow AK, Gonzalez AR. Managing the Planned Cessation of a Global Supply Market: Lessons Learned From the Global Cessation of the Trivalent Oral Poliovirus Vaccine Market. J Infect Dis 2017;216:S40–5. 10.1093/infdis/jiw571 PMC5853836 28838167

[9] John J., Giri S., Karthikeyan A.S., Iturriza-Gomara M., Muliyil J., Abraham A. (2014). Effect of a single inactivated poliovirus vaccine dose on intestinal immunity against poliovirus in children previously given oral vaccine: an open-label, randomised controlled trial. Lancet Lond Engl.

[10] Wright P.F., Connor R.I., Wieland-Alter W.F., Hoen A.G., Boesch A.W., Ackerman M.E. (2016). Vaccine-induced mucosal immunity to poliovirus: analysis of cohorts from an open-label, randomised controlled trial in Latin American infants. Lancet Infect Dis.

[11] Jafari H., Deshpande J.M., Sutter R.W., Bahl S., Verma H., Ahmad M. (2014). Efficacy of inactivated poliovirus vaccine in India. Science.

[12] Asturias E.J., Bandyopadhyay A.S., Self S., Rivera L., Saez-Llorens X., Lopez E. (2016). Humoral and intestinal immunity induced by new schedules of bivalent oral poliovirus vaccine and one or two doses of inactivated poliovirus vaccine in Latin American infants: an open-label randomised controlled trial. Lancet Lond Engl.

[13] Gamage D, Mach O, Palihawadana P, Zhang Y, Weldon WC, Oberste MS, et al. Boosting of Mucosal Immunity After Fractional-Dose Inactivated Poliovirus Vaccine. J Infect Dis 2018;218:1876–82. 10.1093/infdis/jiy389 PMC9161111 29982532

[14] Hird T.R., Grassly N.C. (2012). Systematic review of mucosal immunity induced by oral and inactivated poliovirus vaccines against virus shedding following oral poliovirus challenge. Plos Pathog.

[15] Herremans T.M., Reimerink J.H., Buisman A.M., Kimman T.G. (1950). Koopmans MP (1999) Induction of mucosal immunity by inactivated poliovirus vaccine is dependent on previous mucosal contact with live virus. J Immunol Baltim Md.

[16] Famulare M., Selinger C., McCarthy K.A., Eckhoff P.A., Chabot-Couture G., Riley S. (2018). Assessing the stability of polio eradication after the withdrawal of oral polio vaccine. Plos Biol.

[17] Thompson K.M., Tebbens R.J.D. (2017). Lessons from the polio endgame: overcoming the failure to vaccinate and the role of subpopulations in maintaining transmission. J Infect Dis.

[18] Norton E.B., Lawson L.B., Freytag L.C., Clements J.D. (2011). Characterization of a mutant escherichia coli heat-labile Toxin, LT(R192G/L211A), as a safe and effective oral adjuvant. Clin Vaccine Immunol.

[19] Clements J.D., Norton E.B., Papasian C.J. (2018). The Mucosal Vaccine Adjuvant LT(R192G/L211A) or dmLT. mSphere.

[20] Norton E.B., Bauer D.L., Weldon W.C., Oberste M.S., Lawson L.B., Clements J.D. (2015). The novel adjuvant dmLT promotes dose sparing, mucosal immunity and longevity of antibody responses to the inactivated polio vaccine in a murine model. Vaccine.

[21] White J.A., Blum J.S., Hosken N.A., Marshak J.O., Duncan L., Zhu C. (2014). Serum and mucosal antibody responses to inactivated polio vaccine after sublingual immunization using a thermoresponsive gel delivery system. Hum Vaccin Immunother.

[22] Lee T., Gutiérrez R.L., Maciel M., Poole S., Testa K.J., Trop S. (2021). Safety and immunogenicity of intramuscularly administered CS6 subunit vaccine with a modified heat-labile enterotoxin from enterotoxigenic Escherichia coli. Vaccine.

[23] El-Kamary S.S., Cohen M.B., Bourgeois A.L., Van De Verg L., Bauers N., Reymann M. (2013). Safety and immunogenicity of a single oral dose of recombinant double mutant heat-labile toxin derived from enterotoxigenic Escherichia coli. Clin Vaccine Immunol.

[24] Bernstein D.I., Pasetti M.F., Brady R., Buskirk A.D., Wahid R., Dickey M. (2019). A Phase 1 dose escalating study of double mutant heat-labile toxin LTR192G/L211A (dmLT) from Enterotoxigenic Escherichia coli (ETEC) by sublingual or oral immunization. Vaccine.

[25] Svennerholm A.M., Qadri F., Lundgren A., Kaim J., Rahman Bhuiyan T., Akhtar M. (2021). Induction of mucosal and systemic immune responses against the common O78 antigen of an oral inactivated ETEC vaccine in Bangladeshi children and infants. Vaccine.

[26] Frederick D.R., Goggins J.A., Sabbagh L.M., Freytag L.C., Clements J.D., McLachlan J.B. (2018). Adjuvant selection regulates gut migration and phenotypic diversity of antigen-specific CD4+ T cells following parenteral immunization. Mucosal Immunol.

[27] Maciel M., Bauer D., Baudier R.L., Bitoun J., Clements J.D., Poole S.T. (2019). Intradermal or Sublingual Delivery and Heat-Labile Enterotoxin Proteins Shape Immunologic Responses to a CFA/I Fimbria-Derived Subunit Antigen Vaccine against Enterotoxigenic Escherichia coli. Infect Immun.

[28] Maciel M., Smith M., Poole S.T., Laird R.M., Rollenhagen J.E., Kaminski R.W. (2019). Evaluation of the reactogenicity, adjuvanticity and antigenicity of LT(R192G) and LT(R192G/L211A) by intradermal immunization in mice. PLoS One.

[29] Lewis I, Ottosen A, Rubin J, Blanc DC, Zipursky S, Wootton E., A Supply and demand management perspective on the accelerated global introductions of inactivated poliovirus vaccine in a constrained supply market. J Infect Dis 2017;216:S33–9. 10.1093/infdis/jiw550 PMC5853471 28838159

[30] Okayasu H, Sein C, Blanc DC, Gonzalez AR, Zehrung D, Jarrahian C, et al., Intradermal Administration of Fractional Doses of Inactivated Poliovirus Vaccine: A Dose-Sparing Option for Polio Immunization. J Infect Dis 2017;216:S161–7. 10.1093/infdis/jix038 PMC5853966 28838185

[31] Maciel M., Smith M., Poole S.T., Laird R.M., Rollenhagen J.E., Kaminski R.W. (2019). Evaluation of the reactogenicity, adjuvanticity and antigenicity of LT(R192G) and LT(R192G/L211A) by intradermal immunization in mice. PLoS One.

[32] Javier Martín (Ed.), Poliovirus: Methods and Protocols, Methods in Molecular Biology, vol. 1387, DOI 10.1007/978-1-4939-3292-4_8, © Springer Science+Business Media New York 2016.

[33] Wright PF, Wieland-Alter W, Ilyushina NA, et al., Intestinal immunity is a determinant of clearance of poliovirus after oral vaccination. J Infect Dis 2014;209:1628–34. 10.1093/infdis/jit671 24459191

[34] Van Damme P., De Coster I., Bandyopadhyay A.S., Revets H., Withanage K., De Smedt P. (2019). The safety and immunogenicity of two novel live attenuated monovalent (serotype 2) oral poliovirus vaccines in healthy adults: a double-blind, single-centre phase 1 study. Lancet.

[35] De Coster I., Leroux-Roels I., Bandyopadhyay A.S., Gast C., Withanage K., Steenackers K. (2021). Safety and immunogenicity of two novel type 2 oral poliovirus vaccine candidates compared with a monovalent type 2 oral poliovirus vaccine in healthy adults: two clinical trials. Lancet.

[36] Sáez-Llorens X., Bandyopadhyay A.S., Gast C., Leon T.D., DeAntonio R., Jimeno J. (2021). Safety and immunogenicity of two novel type 2 oral poliovirus vaccine candidates compared with a monovalent type 2 oral poliovirus vaccine in children and infants: two clinical trials. Lancet.

[38] Samuel B.U., Cherian T., Sridharan G., Mukundan P., John T.J. (1991). Immune response to intradermally injected inactivated poliovirus vaccine. Lancet.

[39] Samuel B.U., Cherian T., Rajasingh J., Raghupathy P., John T.J. (1992). Immune response of infants to inactivated poliovirus vaccine injected intradermally. Vaccine.

[40] Nirmal S., Cherian T., Samuel B.U., Rajasingh J., Raghupathy P., John T.J. (1998). Immune response of infants to fractional doses of intradermally administered inactivated poliovirus vaccine. Vaccine.

[41] Yoshino N., Kanekiyo M., Hagiwara Y., Okamura T., Someya K., Matsuo K. (2010). Intradermal delivery of recombinant vaccinia virus vector DIs induces gutmucosal immunity. Scand J Immunol.

[42] Enioutina E.Y., Visic D., Daynes R.A. (2000). The induction of systemic and mucosal immune responses to antigen-adjuvant compositions administered into the skin: alterations in the migratory properties of dendritic cells appears to be important for stimulating mucosal immunity. Vaccine.

[43] Dietrich J., Andreasen L.V., Andersen P., Agger E.M., Rodrigues M.M. (2014 Jun 23). Inducing dose sparing with inactivated polio virus formulated in adjuvant CAF01. PLoS One.

[44] Lundgren A., Bourgeois L., Carlin N., Clements J., Gustafsson B., Hartford M. (2014). Safety and immunogenicity of an improved oral inactivated multivalent enterotoxigenic Escherichia coli (ETEC) vaccine administered alone and together with dmLT adjuvant in a double-blind, randomized, placebo-controlled Phase I study. Vaccine.

[45] Jorba J., Diop O.M., Iber J., Henderson E., Zhao K., Quddus A. (2019). Update on Vaccine-Derived Poliovirus Outbreaks — Worldwide, January 2018–June 2019. Mmwr Morbidity Mortal Wkly Rep.

[46] Brickley EB, Strauch CB, Wieland-Alter WF, et al., Intestinal Immune Responses to Type 2 Oral Polio Vaccine (OPV) Challenge in Infants Previously Immunized With Bivalent OPV and Either High-Dose or Standard Inactivated Polio Vaccine. J Infect Dis 2018;217:371–80. 10.1093/infdis/jix556 PMC5853416 29304199

[47] Brickley E.B., Connor R.I., Wieland-Alter W.F., Collett M.S., Hartford M., Van Der Avoort H. (2019). Intestinal antibody responses to a live oral poliovirus vaccine challenge among adults previously immunized with inactivated polio vaccine in Sweden. Bmj Global Heal.

[48] Senda T., Dogra P., Granot T., Furuhashi K., Snyder M.E., Carpenter D.J. (2019). Microanatomical dissection of human intestinal T-cell immunity reveals site-specific changes in gut-associated lymphoid tissues over life. Mucosal Immunol.

[49] Thome J.J.C., Bickham K.L., Ohmura Y., Kubota M., Matsuoka N., Gordon C. (2016). Early-life compartmentalization of human T cell differentiation and regulatory function in mucosal and lymphoid tissues. Nat Med.

[50] Crespo M., Martinez D.G., Cerissi A., Rivera-Reyes B., Bernstein H.B., Lederman M.M. (2012). Neonatal T-cell maturation and homing receptor responses to Toll-like receptor ligands differ from those of adult naive T cells: relationship to prematurity. Pediatr Res.

[51] Connor R.I., Brickley E.B., Wieland-Alter W.F., Ackerman M.E., Weiner J.A., Modlin J.F. (2022). Mucosal immunity to poliovirus. Mucosal Immunol.

[52] Din M., Asghar M., Ali M. (2020). Delays in polio vaccination programs due to COVID-19 in Pakistan: a major threat to Pakistan’s long war against polio virus. Public Health.

