# Corrigendum to “Intradermal fractional-dose inactivated polio vaccine (fIPV) adjuvanted with double mutant Enterotoxigenic *Escherichia coli* heat labile toxin (dmLT) is well-tolerated and augments a systemic immune response to all three poliovirus serotypes in a randomized active-controlled trial” [Vaccine 40(19) (2022) 2705–2713]

**DOI:** 10.1016/j.vaccine.2022.05.001

**Published:** 2022-05-31

**Authors:** Jessica W. Crothers, Elizabeth Ross Colgate, Kelly J. Cowan, Dorothy M. Dickson, MaryClaire Walsh, Marya Carmolli, Peter F. Wright, Elizabeth B. Norton, Beth D. Kirkpatrick

**Affiliations:** a Department of Pathology and Laboratory Medicine, Vaccine Testing Center, University of Vermont Larner College of Medicine, Burlington, VT, USA; b Microbiology and Molecular Genetics, Vaccine Testing Center, University of Vermont Larner College of Medicine, Burlington, VT, USA; c Department of Pediatrics, Vaccine Testing Center, University of Vermont Larner College of Medicine, Burlington, VT, USA; d Department of Pediatrics, Geisel School of Medicine at Dartmouth, Hanover, NH, USA; e Department of Immunology and Microbiology, Tulane University, New Orleans, LA, USA

The authors regret that that the title of their published work requires an update. While we utilized the term “placebo-controlled” in our original title and methods, we believe that “active-controlled” trial more accurately describes the design of our study. Subjects randomized to the control arm received a fractionated dose of intradermal IPV without the mucosal adjuvant (dmLT) under study, making their designation as a placebo arm inappropriate. This is an important distinction which we feel significantly impacts both interpretation of the resultant data and any conclusions of the study. We apologize that this was not noticed prior to publication and the revised title is now reproduced above.

The authors would like to apologise for any inconvenience caused.

