# Supplementary material for: Intradermal fractional-dose inactivated polio vaccine (fIPV) adjuvanted with double mutant Enterotoxigenic Escherichia coli heat labile toxin (dmLT) is well-tolerated and augments a systemic immune response to all three poliovirus serotypes in a randomized placebo-controlled trial
Source: Vaccine. 2022 Apr 26;40(19):2705–13. doi: 10.1016/j.vaccine.2022.03.056 (PMC9024222; doi:10.1016/j.vaccine.2022.03.056)
Supplement: Supplementary data 1 [file mmc1.docx]

**Supplementary Table 1. Serotype Specific Fecal IgA by Day and Treatment Group**

|  |  | fIPV only (N=10) | | | fIPV+dmLT (N=19) | | | P value* |
| --- | --- | --- | --- | --- | --- | --- | --- | --- |
|  | Day | q1 | median | q3 | q1 | median | q3 |  |
| Total IgA | 0 | 12278 | 31839 | 221335 | 7206 | 41806 | 72718 | 0.84 |
|  | 28 | 13216 | 40657 | 137204 | 4075 | 7011 | 176996 | 0.54 |
|  | Change | -32237 | 1081 | 119904 | -34946 | -1923 | 144736 | 0.95 |
| MFI PV1 | 0 | 1.75 | 10 | 22 | 5 | 20 | 31 | 0.29 |
|  | 28 | 15 | 24.5 | 29.75 | 8.5 | 16 | 20.5 | 0.25 |
|  | Change | 2.5 | 10.5 | 18 | -14 | 1 | 12 | 0.14 |
| MFI PV2 | 0 | 9.25 | 14 | 36.25 | 12.5 | 20 | 39 | 0.26 |
|  | 28 | 25 | 35.5 | 46.5 | 18 | 26 | 37 | 0.23 |
|  | Change | -18 | 8.25 | 17.5 | -10 | 7 | 26 | 0.31 |
| MFI PV3 | 0 | 18.25 | 33.5 | 45.5 | 13.5 | 30 | 56 | 0.98 |
|  | 28 | 18 | 33.5 | 47 | 19 | 30 | 51.5 | 0.95 |
|  | Change | -19.75 | 10.5 | 19.5 | -14.5 | -7 | 15.5 | 0.68 |

* Significance assessed by Mann Whitney U test and p values <0.05 were considered significant.
